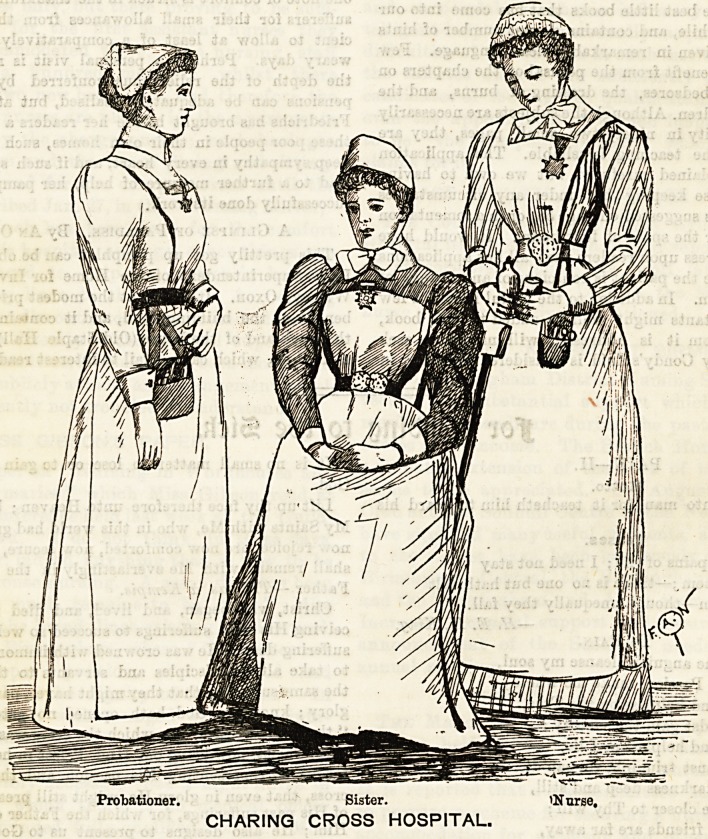# The Hospital Nursing Supplement

**Published:** 1895-02-16

**Authors:** 


### "The Hospital" Nursing Mirror.

Being the Extra Nursing Supplement of "The Hospital" Newspaper.
[Contributions for this Supplement should be addressed to the Editor, The Hospital, 428, Strand, London, W.C., and should have the word "Nursing" plainly written in left-hand top corner of the envelope.]

### News from the Nursing World.

### CONTEMPLATED IMPROVEMENTS.

There is a prospect of the nursing staff at St. George's Hospital being better lodged ere long, certain important additions, including some rebuilding of present quarters, having been sanctioned by the committee and governors, who are willing to grant £18,000 for the purpose. The numerous nurses' homes, which have sprung up in various parts of the country, serve as valuable object lessons from which hospital committees can learn what to avoid, and note what is most worthy of imitation.

### THE LEWISHAM GUARDIANS.

The letter from the Local Government Board, which was laid before the Lewisham Guardians last week, appears to have been received by them with some dissatisfaction. After a discussion it was decided that a deputation, consisting of all the guardians, should wait upon the Local Government and explain the reasons of their unwillingness to follow the recommendations made to them. The most important of these measures, namely, the reinstating of the matron, has been, therefore, postponed until after the deputation has been received.

### THE MILDMAY MISSION HOSPITAL.

The work of the Mildmay Hospital is by no means restricted to the nursing of some fifty in-patients, for a certain number of out-patients, whom the resident medical officer treats in their own homes, are also nursed by the institution. This secures training in district work for missionaries and others desirous of becoming more useful women. We are glad to find that the able superintendent never permits those workers who are received for short terms of instruction and experience to be called nurses. This title is given exclusively to those who go through the full term of training. The head of each ward is a fully-trained sister, competent to instruct probationers, and the latter are also permitted to attend the courses of lectures given to the nursing staff of the London Hospital, a privilege which is highly appreciated. The Mildmay Hospital is extremely well planned as to details, and in most respects the arrangements leave nothing to be desired. All pupils go there with the intention of following permanently some branch of nursing or missionary work. Those employed in district nursing have quarters separate from the nurses engaged in the wards, very comfortable provision being made for all members of the staff. The dispensary is an ideal one, and instruction is given by a qualified lady who has already presided for some years over this department. Cleanliness, order, and method are combined with a pervading spirit of kindliness in this pretty hospital. Most of the excellent furniture was given to the wards, and doubtless the additional funds needed for completing the front, for perfecting the lift, and for painting unfinished portions of the structure, will be eventually supplied by friends of the Mildmay Institution.

### A TRAINING HOSPITAL.

The nursing methods in vogue at the Tottenham Evangelical Protestant Deaconesses Institution would be criticised with more hesitation if the words "Training Hospital" did not also form part of the title of this imposing-looking building. The sitting and dining-rooms are sufficiently pleasant, tables being set for meals in an orderly fashion. Small rooms for senior workers contain from one to three beds, but the juniors share one dormitory, in which no screens nor curtains appear to provide even partial privacy for the occupants. The wards for paying patients are very nice indeed, but, unfortunately, there is little demand for them, and their unoccupied condition is in striking contrast to the somewhat crowded general wards. The kitchens for the latter are small, and shelves for ointments, bottles, &c., look conspicuously out of place on the walls. The surgical dressing-tables seem to live out in the passages, and the attic-like operating theatre has a shaft (the patients' lift) opening right into it. Lectures are given to the nurses by the house surgeon during their training, which is said to last for varying periods of time. As an E.P.D.I., this pleasantly-situated house may be altogether satisfactory, but as a training school it can hardly be considered to meet the requirements of modern skilled nursing.

### WHAT DOES A BADGE GUARANTEE?

Medals and badges possess considerable value as decorations over and above their primary object of showing that their owners are distinguished for specia ability or heroism, or for being associates of some order. But there is a grave danger in accepting such decorations in the light of certificates of character. A tradesman at Melksham is recently reported to have supplied goods on credit to Mary Elizabeth McGregor, who wore the badge of a Nursing Association, and was eventually committed to trial at the next quarter sessions. According to the local press, her connection with the association ceased in June last.

### PAUPERISED OR PROTECTED?

There appears to be much difference of opinion at Kidderminster regarding the nursing of the sick poor. At the last meeting of the Guardians a majority negatived the motion that certain subscriptions should be continued to the institutions at Kidderminster, Stourport, Bewdley, and Wribbenhall, by which the attendance of skilled nurses has been secured to patients receiving outdoor relief from the union. The guardian who seconded the defeated motion drew attention to the obvious economy of granting the services of a nurse to the sick poor in their own homes, yet an opponent of the scheme is reported to have said, "They were putting a premium on pauperism by providing nurses in that way." It is difficult to see how the recipient of a daily visit from a nurse can be as effectively "pauperised" as the sufferer, driven into "the house," when this humane assistance is withheld.

### DIFFICULTIES IN DISTRICT NURSING.

An exhaustive inquiry was lately held by the Oxford city coroner respecting the death of a woman at Summerton. It was suggested in the district that there had been delay in securing a doctor, but all the witnesses agreed as to the frequent attendances made by the nurse, and her attempts to get a medical man, who unfortunately was engaged in another direction with another confinement case. The nurse herself was obviously overworked, having been up during the previous night, but notwithstanding this she is reported to have bestowed adequate care upon the woman, whose serious condition she seems to have realised on first arriving at the case.

### PROVISION FOR BARRY.

The annual general meeting of the Barry District Nursing Association was held last week, and a most creditable balance-sheet was presented to the subscribers. The work of the district nurses has been greatly appreciated, over eighteen thousand visits having been paid to 759 patients. Of these 104 were accident cases, which emphasises the need for the cottage hospital or accident ward, to which we recently referred, as under contemplation. It was agreed by the meeting that the association should take over the new work, and doubtless Barry will be permanently benefited by this decision.

### VACCINATION VIGOROUSLY ADVOCATED.

A letter from the Most Rev. Dr. Walsh, Archbishop of Dublin, on the necessity of vaccination and re-vaccination, was read at the last Mass on a recent Sunday, in all the Roman Catholic churches in Dublin. His Grace censured in the strongest terms the neglect of these precautions, and concluded his practical address by intimating that during the continuance of the present epidemic no unvaccinated person, whether child or adult, can be admitted to confirmation, or to preparatory instruction throughout the diocese.

### HARD LIVES.

Much interest has been taken in the accounts which have from time to time appeared in our columns of the work done by the hospital ship sent to Labrador under the auspices of the Mission to Deep Sea Fishermen. The two hospitals erected respectively at Battle and Indian harbours are much appreciated during the fishing season by patients, many of whom there first make acquaintance with the comforts of spring beds and good blankets. They are all terribly ill-fed and ill-clothed, and their poverty therefore makes the fishing season too important to the men and women it employs for aught save acute illness or accident to reconcile them to giving up work. Thus the inmates of the hospitals are such as need good nursing, in addition to medical attendance, drugs, and nourishment. These have been secured to them by the mission, and the two trained nursing sisters go out in May to spend their third season amongst the poor fishers and their families on the bleak Labrador coast. Strictly necessary things are provided for the hospital wards, but these need supplementing by a few luxuries, such as pictures, well-printed books, and bright chintz or Turkey red for covering screens. Framework for the latter is also needed, for the rooms have often to serve several purposes, and the gales on that bleak coast make protection against draughts requisite. A very little help from some readers interested in medical and nursing work on the Labrador would add materially to the comfort of these two sister-nurses, besides showing the sick folks that workers in the England, which none of them have seen but all dream of, are not unmindful of their lives of hardship.

### A PARSEE HOSPITAL.

The Parsee Maternity Hospital opened last month by Lord Harris is a most valuable addition to the institutions of Bombay. A temporary hospital has been in use there for some time, and has enabled Dr. Temuljee to demonstrate the practical advantages of sanitary over insanitary surroundings. Formerly the dampest and lowest room in a house was considered suitable for the Parsee woman, but modern teaching has ensured fitter provision for her. The Parsees are to be congratulated on the intelligent encouragement they have given to the excellent instructions of Dr. Temuljee.

### SHORT ITEMS.

The annual meeting of the Oldham Nursing Association was well attended, the Mayor acting as chairman.—The Cornwall Trained Nurses' Home at Truro needs increased support from doctors and residents to improve its financial position.—H.R.H. the Duchess of Connaught presented certificates to the members of a nursing class held at Bagshot, under the Surrey County Council Technical Education scheme.—The necessity for improvement in the quarters of the nurses at the Edinburgh Fever Hospital seems to be generally acknowledged.—The Rothesay District Nursing Association, of which the Marchioness of Bute is president, has become affiliated with the Queen's Jubilee Institute.—The Royal British Nurses' Association will in future publish official reports solely in its own organ, the *Nurses' Journal*, which is issued quarterly; any matters of general interest relative to the association will no longer be communicated exclusively to any one paper, but will be found in such journals as devote space to nursing affairs.—A silver medal has been awarded by the French Government to Madame Morel, who has devoted twenty-five years to the sick having done specially valuable work during two small-pox epidemics. A similar decoration has been conferred on Madame Dallery for services during an epidemic of typhoid at Dey, in Algiers.—The Islington Guardians are said to think of appointing a female sanitary inspector, at a salary of £100, to be raised £10 per annum until the maximum of £150 is attained.—A trained district nurse will shortly be installed at Teignmouth, where a nursing association has been recently organised.—The Duchess of Abercorn presided at the annual meeting of the Londonderry District Nursing Society, there was a large attendance.—The inquiry into the alleged neglect of patients in Athlone Union Infirmary brought to light the fact that it has been customary to leave an untrained pauper girl in sole charge of 50 or 60 patients during the night, although four Sisters of Mercy were employed as nurses during the day,

### Elementary Anatomy and Surgery for Nurses.

By W. McAdam Eccles, M.B., M.S., F.R.C.S., Assistant Surgeon and Lecturer to Nurses, West London Hospital.

### VI.—PREVENTION OF SUPPURATION. ANTISEPTIC AND ASEPTIC SURGERY.

Unseen foes are always more difficult to cope with than those which meet us openly. Suppuration, or the formation of pus, is produced by the action of agents hidden to the naked eye, known now to be parasitic vegetable micro-organisms for the most part. It becomes, therefore, of the utmost importance for all concerned in the treatment of wounds to thoroughly master the methods whereby these annoying bacteria may be excluded from the tissues.

A patient's wound may be infected from various sources. First and foremost of these is the patient's own skin; next, the hands of the surgeon and his assistants, the latter including the nurse; then the instruments used; and, lastly, the surroundings of the patient. We will take these in detail.

### The Patient's Skin.

Nothing is, perhaps, more septic than the skin itself. Here we must pause to define some terms which will be in constant use throughout this lecture. By *sepsis* we understand the contamination of a wound with putrefactive or infective bacteria, and a part is termed aseptic when no micro-organisms likely to lead to the formation of pus are present. Operations performed in such areas constitute aseptic surgery. A region may be rendered aseptic either by the removal or death of all the bacteria which may have been present. Thorough cleansing may produce the former, but an antiseptic, *i.e.*, a chemical substance which acts against septic organisms, will bring about the latter. Thus it will be observed that there is a distinct difference between the terms asepsis and antisepsis, the first implying the entire freedom from pus-producing bacteria, the second the effort by the use of reagents to render a part aseptic. We have now to set about preparing the skin of a patient, so that at the appointed time the area for the operation may be aseptic.

Let us employ both the means at our disposal, that of attempting to remove all extraneous matter, and failing in this, to apply an antiseptic to destroy any living germ. It must be borne in mind that not only the surface of the skin is "dirty," but what is far more important to realise, that the depths of the glands which open on the surface by the pores are crowded with our hidden foes. Soap and water is our first friend and ally. Let the patient, if feasible, have a warm bath the day before the operation. The skin if necessary should be shaved, and then thoroughly scrubbed with soap and water and a piece of flannel or lint, and not merely delicately washed. The effect of this application should be a very distinct reddening of the skin. If this process can be performed two days before the operation and repeated, so much the better, I am convinced, will be the result.

The skin has naturally a certain amount of greasy or oily matter secreted upon it, and this being septic in its nature requires to be removed. Soap and water may not be sufficient to effect this, therefore, it is advisable to employ some turpentine or ether, either of which has the action of dissolving grease. It is well to remove the excess of these by water which has been boiled, and thus rendered sterile, that is, has had all living organisms in it destroyed. The area of skin which has been cleansed thus should be thoroughly washed over with one of the various efficient antiseptic solutions. Of the many in use perhaps three may be stated to be most commonly employed.

First, there is a strong poison known as the perchloride of mercury, or corrosive sublimate. This is so powerful a germicide that a solution of 1 part in 1,000 parts of boiled water is amply sufficient (*i.e.*, 10 grains to one pint of water). Secondly, carbolic acid is very frequently applied in the strength of 1 in 20, or 1 in 30 (*i.e.*, one ounce of liquid carbolic acid to one pint of boiled water, or one ounce to one and a-half pints of boiled water). Lastly, a solution of permanganate of potassium (Condy's fluid) may be preferred (*i.e.*, 4½ grains to an ounce of boiled water). After this application has been made, the part should be covered with an antiseptic dressing—such as lint or gauze, which has been impregnated with an antiseptic; and at the time of its being placed on the skin it is moistened with an antiseptic solution. Sal alembroth gauze ("blue" gauze), which is gauze which has been treated with perchloride of mercury, or carbolic gauze answers well. The dressing should be securely fixed in position by a gauze bandage, and left undisturbed until removed at the time of the operation. Such is the outline of the method I employ for the cleansing of the skin of the operation area, and also that of some distance around it.

### The Hands of the Surgeon and his Assistants.

These must be rendered as thoroughly clean as the skin of the patient, and in practically a similar manner. Much attention must be paid to the proper purification of the parts about the nails; a clean nail-brush should always be employed. Nurses particularly should be careful to render their hands aseptic before assisting at an operation.

### The Instruments Used.

All instruments should if possible be boiled just before use at an operation. Sponges are perhaps the most treacherous of the various articles employed. New sponges, after being freed from all extraneous matter, as sand, by beating and then soaking in repeated changes of water, are then treated with a dilute solution of sulphurous acid (1-5 is the best strength). They are afterwards to be well rinsed in boiled water, and either kept in a solution of 1-40 carbolic acid lotion until use, or steeped in a 1-20 carbolic solution for twenty-four hours and then squeezed dry and kept in air-tight bottles or tins. After use in an operation, provided no pus was present, they should be thoroughly washed in warm water—hot water, much less boiling water, should never be used—and then placed in a solution of ordinary washing soda for about twelve hours. After this they should be well rinsed in boiled water, and treated with carbolic lotion as above. If contaminated by pus they are best destroyed.

### The Surroundings of the Patient.

The room in which the operation is to be undertaken must be very clean and airy. All the region around the area of the operation should be covered with mackintosh sheeting, overspread with towels rung out of 1-20 carbolic solution. Nothing must come in contact with the wound which is not surgically clean, that is, aseptic.

If the above details be fully carried out, very gratifying results will follow in the way of wounds healing without a drop of pus. This success is known as aseptic surgery, and this should be the aim of both surgeon and nurse in all possible cases.

### Appointments.

[It is requested that successful candidates will send a copy of their applications and testimonials, with date of election, to The Editor, The Lodge, Porchester Square, W.]

Dundonald Convalescent Home, Kilmarnock.—Miss M. A. Barlas has been appointed Matron of the Dundonald Convalescent Home. She was trained at the Children's Hospital, Birmingham; the Victoria Infirmary, Glasgow; the Radcliffe Infirmary, Oxford; and was for three years district nurse in Gloucester. We wish her every success in her new appointment on which we congratulate her.

### Probationers and Junior Nurses.

By Nurse Mildred.

Some time ago I wrote in The Hospital columns on preparations for hospital life, and now I address those who have made a start, but yet are new and strange, and possibly unhappy. All begin with similar feelings; they will pass with you, as with others, when you are no longer "new probationers." Nobody ever really forgets the first day in hospital, although impressions get less distinct, and we are not as sympathetic or considerate as we might be to those who come after us.

This hospital world is a strange one, different from anything experienced before. You are awkward, and you know it; and it is humiliating, after having performed creditably the duties of life, to suddenly find yourself beginning again with everything to learn, from sometimes impatient teachers.

Two resolutions are needful to start with; to give unquestioning obedience to those over you, and to be forbearing if blamed unreasonably. A quiet, respectful answer, or sometimes no answer at all "turneth away wrath." Put yourself in the sister's or staff nurse's place, when a new probationer comes in place of a nurse who, in hospital parlance, has "got into sister's ways."

It is not your fault that you know nothing, for you have come to be trained. At the same time, in the rush of ward work (possibly heavier than in the previous week when a more experienced nurse assisted) tempers get ruffled, which is not justifiable, of course; but, thinking of the difficulties, make such excuses as you can. Space does not admit of my giving strictly nursing hints, and there are many good text-books which you can study; and, besides, you are in the best place to learn if you keep ears and eyes open and ask questions of those who can answer them, remembering to choose suitable times for your queries.

I want particularly to suggest the moral qualification essential from the beginning on to the end of the trained nurse's life. The necessity for accuracy, strict truthfulness, sinking *amour propre* before the demands of conscience, and the welfare of patients. Patience and self-control under provocation, perseverance under discouragement, unselfishness, self-abnegation—all these are wanted.

A few other points, to my mind very important, are, first, the duty of loyalty to those placed over you. This duty is too often forgotten, though inculcated in early times on the highest authority. Where there is affection, the outcome of knowledge of a person's character, and trust in that person, it is easy; but at the outset we have not these helps, and yet we must be loyal. Loyalty consists not only in refraining from open grumbling or insinuations, but also in using our influence against insubordination. If a grievance exists or one is imagined, go to the right person honestly and have the small wrongs righted, remembering that in an institution the good of the whole must be considered before the comfort and convenience of the individual, if the interests clash. In a large community there are always malcontents, sometimes with reason, sometimes without. Loyalty does not always meet with a reward. You will be disliked and unpopular, perhaps taunted with "toadying" to those in authority, but the reward of "a good conscience" is worth a great deal.

Cultivate true courtesy—that is, consideration for others' feelings. Cut off from home and social duties manners are apt to deteriorate; but in hospital life the amenities of society should not be ignored. I would also insist on the advantages of habitual punctuality, not only in the order and routine of the wards, where sisters will not tolerate nurses coming systematically late on duty, but in instances where the rule is not so strictly enforced and where unpunctuality means self-indulgence. Nurses sometimes say, "I am never unpunctual when it really matters"; but when does it not "really matter"?

Again, hospital etiquette is strange and irksome to beginners; but it is necessary in maintaining order. The relative positions of nurses and their superiors in hospitals are, of course, quite arbitrary, having nothing to do with social position. Respect is due to those more advanced in professional knowledge and experience than yourselves. Failure to understand this shows want of perception.

Now for a word about personal health. Don't be fidgetty over trifling ailments, forget them; but, at the same time, always own promptly to feeling ill. It is better for yourself and everybody else that you should be treated in time. Do not doctor yourself. Observe common-sense rules for keeping in health—going to bed early, being careful about personal cleanliness and nourishment, and going out regularly.

If physically tired on coming off duty, part of your time may very well be spent lying down, provided the rest be spent in the fresh air, whether it be wet or fine, otherwise you will risk hospital throats, bad fingers, &c.

In your busy daily work do not let real religion pass out of your life, for it cannot be beautiful or complete without that. Some nurses live like heathens, whilst others make great profession often without practice. What is wanted for hospital workers is reality, not "make believe."

Let your practice be equal to your profession, otherwise the harm done to religion amongst those around you may be incalculable and your responsibility is great in this matter.

### Where to Go.

A lecture by Dr. Bezley Thorne on "Physical Treatment of Heart Diseases" will be substituted for the one previously announced for Friday, February 15th, at the offices of the Royal British Nurses' Association, 17, Old Cavendish-street, at eight p.m.

Cookery and Food Exhibition.—The Universal Cookery and Food Exhibition is to be held in Portman Rooms, Baker Street, May 7th to 10th. Half of the profits will be devoted to providing meals for poor children in the metropolis. The offices of the exhibition are at 329, Vauxhall Bridge Road, S.W.

Greg Memorial Hall, 8, Dawson Street, Dublin.—At half-past three p.m. on Tuesday, February 19th, the annual meeting of St. Patrick's Nurses' Home, affiliated with Q.V.J. Institute, will take place, Archbishop of Dublin in the chair; first resolution to be proposed by Ven. W. Sinclair, D.D., Archdeacon of London.

### Notes and Queries.

**Queries.**

(76) *Books.*—What books would be helpful to a probationer entering a London hospital? Is Hoblyn's Dictionary good?—*G. R.*

(77) *Insurance.*—Where can I get information about insurance against accident and illness?—*M. D.*

(78) *Florence.*—How can I get particulars as to the Hospital for Sick Babies at Florence?—*L. O. S.*

(79) *Nurses' Co-operation.*—I shall be much obliged by information as to whether this association has changed its address, or if it has any branches in London. I thought the title belonged exclusively to the establishment at 8, New Cavendish Street, but I see it is displayed elsewhere.—*Nurse Janet.*

**Answers.**

(76) *Books* (*G. R.*).—You would find Hoblyn's "Dictionary of Medical Terms" most useful for reference; also Lewis's "Theory and Practice of Nursing," and Miss Lücke's "Lectures on Nursing." Write for catalogue to the Scientific Press, 428, Strand, London.

(77) *Insurance* (*M. D.*).—Write to British Medical Association, at 434, Strand.

(78) *Florence* (*L. O. S.*).—By writing direct to the Superintendent.

(79) *Nurses' Co-operation* (*Nurse Janet*).—The Nurses' Co-operation is at 8, New Cavendish Street, and has no branches. You should write or call on the Lady Superintendent.

### Wants and Workers.

[The attention of correspondents is directed to the fact that "Helps in Sickness and to Health" (Scientific Press, 428, Strand) will enable them promptly to find the most suitable accommodation for difficult cases.]

Can any reader of The Hospital please tell a nurse where she could get a second-hand wicker bath-chair with rubber tyre wheels and self-guiding front wheel, cheap for cash, must be in fair condition, for a patient who is not well off?—*Nurse Alice.*

### Dress and Uniforms.

By a Matron and Superintendent of Nurses.

### CHARING CROSS HOSPITAL.

Visitors to Charing Cross Hospital cannot fail to be charmed with the neat and effective uniform worn by its nursing staff. Variety, too, is apparent, not only in the distinctive dress of the sister, staff nurse, and probationer, but in the shape of their respective caps. Navy blue sateen is worn by the sisters, a material which has the advantage of being washable, an important consideration in these days of antiseptics. The skirt, which just clears the ground, is turned up with a deep hem, headed with a couple of narrow tucks stitched with red. The apron is made of jaconet, with a wide hem round the bottom, and a square bib. Neat linen cuffs give a finish at the wrist to a rather full sleeve, and a cap of the shape with which Sister Dora has made us so familiar, ties with narrow muslin strings under the chin, completing the costume.

The staff nurses' dress is blue and white striped galateal with an alternate stripe of red which gives a brightness to the uniform, and at the same time redeems it from being common-place. The apron, like that worn by the sisters, is of jaconet, but has straps attached to the bib which cross at the back and fasten at the waist. The sleeves are very full, but fit close towards the wrist to admit of the cuff being worn over them. Especially pretty is the cap which is shaped neatly to the head, and finished off with two rows of gophered lace frilling, with plain muslin strings that tie in a neat bow in front.

Linen, of the soft delicate shade known as *vieux rose*, is the dress worn by the probationers, and very fresh and cheerful in appearance it is. The apron is made of calico with bib and straps which fasten at the back. A square of muslin, edged with lace, is arranged to form the cap, one of the points coming in front, and the others pinned into position at the back. Plain linen cuffs and collars give the finishing touches to each of the costumes described.

Blue Imperial Russian cloaks are worn by the nurses out of doors, those of the sisters being distinguished by a red silk facing.

Probationer. Sister. Nurse.

CHARING CROSS HOSPITAL.

### DONEGAL LINENS.

At the Booksellers' Exhibition at St. Stephen's Hall, Westminster, Mrs. Ernest Hart held a most attractive stall devoted to Donegal linens used as book coverings. The colours and textures of these linens are well known and appreciated, and the idea of adapting them for book bindings is a most happy one. The effect is exceedingly pretty either plain or ornamented by painting or needlework. Some painted in Japanese designs afforded an excellent suggestion for a novelty in bindings for editions de luxe, and for blotters and a variety of other articles. At the exhibition the show was naturally limited to suitable exhibits, but we can well imagine how well the pretty Donegal linens would lend themselves to the covering of photograph screens and frames, the colours being excellent background for effective painted designs. We think our readers would find a visit to the Irish depôt in Wigmore Street both interesting and profitable.

### The Book World for Women and Nurses.

[We invite Correspondence, Criticism, Enquiries, and Notes on Books likely to interest Women and Nurses. Address, Editor, THE HOSPITAL (Nurses' Book World), 428, Strand, W.C.]

BOOKLAND AND ITS INHABITANTS. By JOHN ENNAR. (Published by F. L. Ballin, 5, Agar Street, Strand. Price 1s )

In an amusing little volume, illustrated by eight sketches, the work of Alice Rivett-Carnac, Mr. Ennar summarises the weaknesses of the average novel. Many of the characters satirised are familiar to readers of romance, and, if he deals somewhat hardly with our favourite heroes and heroines, he only puts into words the disillusions which are, alas! an inevitable part of growing up!

HOW TO NURSE IN OUR OWN HOMES. By A. M. ALEXANDER. (Published by Wells Gardner, Darton, and Co., 3, Paternoster Buildings, and 44, Victoria Street. Price 6d. in cloth and 3d. in paper cover.)

This is one of the best little books that has come into our hands for a long while, and contains a vast number of hints on home nursing given in remarkably clear language. Few people can fail to benefit from the perusal of the chapters on the prevention of bedsores, the dressing of burns, and the nursing of sick children. Although the subjects are necessarily treated with brevity in ninety-two small pages, they are treated well, and the teaching is reliable. The application of poultices is explained carefully, but we own to having little faith in these keeping hot under any circumstances for twelve hours, as suggested on page 58, or in a fomentation being of benefit for the space of four hours. It would have been better to impress upon readers that all hot applications need renewal before the patient is conscious of any change of temperature in them. In addition to the useful index, a few facts about disinfectants might well be added to this book, for those to whom it is addressed will naturally seek for the reason why Condy's fluid is considered sufficient in one case while carbolic is required in another. Perhaps the author may contemplate some slight additions to the next edition of this excellent book. It is one of the best and safest little guides that we have ever come across, and should be widely distributed.

BENT, NOT BROKEN. By HULDA FRIEDRICHS.

Miss Friedrichs gives us in this little booklet sadly pathetic glimpses into the lives of some of the pensioners of the Royal Hospital for Incurables. The same tale of long years of patiently borne suffering, and hopeless struggling against the overwhelming odds of ill-health and poverty runs through all its pages with varying details, and in each the one note of comfort is struck in the thankfulness of these poor sufferers for their small allowances from the hospital, sufficient to allow at least of a comparatively peaceful end to weary days. Perhaps a personal visit is necessary before the depth of the relief thus conferred by these outside pensions can be adequately realised, but at any rate Miss Friedrichs has brought before her readers a vivid picture of these poor people in their own homes, such as must arouse deep sympathy in every heart, and if such sympathy should lead to a further measure of help, her pamphlet will have successfully done its work.

A GLIMPSE OF PARADISE. By AN OUTSIDER.

This prettily got up pamphlet can be obtained from the Lady Superintendent of the Home for Invalid Children at Witney, Oxon. It is sold at the modest price of 7d., for the benefit of the building fund, and it contains an account of the work and of the house (Old Staple Hall) in which it is carried on, which cannot fail to interest readers.

### For Reading to the Sick.

### PAIN—II.

**Motto.**

PAIN is useful unto man, for it teacheth him to guard his life.—*M. Tupper.*

**Verses.**

Many are pains of life; I need not stay
To count them;—there is no one but hath felt
Some of them—though unequally they fall.
—*H. E. H. King.*

TO PAIN.

By Thine anguish cleanse my soul,
By Thy Passion, make me whole;
Weak and helpless on the Tree,
Thou didst gain the victory:
Weak and helpless as I lie,
Thou canst triumph, sin can die.
In the darkness deep and still,
Bind me closer to Thy will;
Earthly friends are far away,
Be Thou with me night and day:
Earthly happiness I miss,
Make me conscious of Heaven's bliss.
Make me pure that I may be
Able to be one with Thee,
And reveal Thyself, for Thou
Art the thing I long for now.
When the veil at last is riven,
To behold Thee will be Heaven.
—*Caroline M. Noel.*

**Reading.**

Are not all painful things to be endured for the sake of life Eternal? It is no small matter to lose or to gain the Kingdom of God.

Lift up thy face therefore unto Heaven; behold I and all My Saints with Me, who in this world had great conflicts, do now rejoice, are now comforted, now secure, now at rest, and shall remain with Me everlastingly in the Kingdom of My Father.—*Thomas à Kempis.*

Christ, who began, and lived, and died in sorrows, perceiving His own sufferings to succeed so well, and that "for suffering death, He was crowned with immortality," resolved to take all His disciples and servants to the fellowship of the same suffering, that they might have a participation of His glory; knowing, God hath opened no gate of heaven but "the narrow gate," to which the cross was the key. And since Christ, now being our High Priest in heaven, intercedes for us by representing His passion, and the dolours of the cross, that even in glory He might still preserve the mercies of His past sufferings, for which the Father did so delight in Him; He also designs to present us to God dressed in the same robe, and treated in the same manner, and honoured with "the marks of the Lord Jesus;" "He hath predestinated us to be conformable to the image of His Son." And if under a head crowned with thorns, we bring to God members circled with roses, and softness, and delicacy, triumphant members in the militant Church, God will reject us, He will not know us who are so unlike our elder Brother.
—*Jeremy Taylor.*

Jesus Christ the Son of God sanctified Pain for ever by His cross. If Christ had not suffered and died would He have drawn all men unto Himself? The suffering King has, strange to say, a majesty and dignity greater than that of all the conquerors of the world. And why? Because He showed the exceeding beauty and nobleness of Sacrifice, of enduring pain and anguish that others might gain.—*C. M. Hallett.*

### Our American Letter.

Communicated.

Before this letter is in type the meeting of the Superintendents at Boston will be over. Two conferences are arranged for 13th, and two for the 14th inst., a meeting of the council being announced for the evening of 12th. We are hoping for a gathering of superintendents from most of our hospitals and expect that many subjects of interest will be discussed.

On New Year's Day the formal opening of the Seton Hospital for Consumptives took place at Spurgten Duyvil, the medical superintendent being Dr. J. W. Roosevelt, whilst the nursing is under the direction of Sister Marie Dolores.

The Josephine Training School at Chicago gives a two years' course to probationers, who have each week two lectures from members of the medical staff. The practical training is received in the Charity, the Post Graduate, and the Chicago Hospitals, and the graduating exercises on January 2nd formed an occasion for a large reception, many friends of the nurses being present and a distribution of diplomas and badges taking place. An alumnæ association has been formed in connection with the Josephine Training School.

To the Winnipeg Training School, which is associated with the General Hospital, a second nurses' home has been erected. This is a separate building devoted to the nurses in charge of the infectious wards. The lady superintendent of this hospital is Miss B. Holland.

Mrs. Zevorick, who was once a graduate of Boston City Hospital, has been instrumental in organising a district nursing society at San Francisco, and already good work has been done for the sick poor in their own homes.

An interesting novelty was introduced into the commencement exercises at the Philadelphia Women's Hospital. This consisted in an exhibition of specimens of the sick cookery practised by the graduates in the diet kitchen, and the dishes were excellently served. They included all kinds of foods suitable for sick and convalescent patients, besides a variety of invalid drinks.

The long-talked of City Hospital car has recently been completed at St. Louis, the trial trip having been made soon after Christmas, it runs on the rails of the usual tram cars.

### Everybody's Opinion.

[Correspondence on all subjects is invited, but we cannot in any way be responsible for the opinions expressed by our correspondents. No communications can be entertained if the name and address of the correspondent is not given, or unless one side of the paper only be written on.]

### NURSE OR WASHERWOMAN.

"One of the Guardians" writes: Under this title in your issue of January 26th you seem to be under the impression that the nurse at the Aylesbury Union House was equally employed in nursing and getting up the wash, and perhaps the discussion in the board-room may have given some reason for this. Doubtless the nurse in the past occasionally in her leisure assisted in the laundry, but this was not by the direction or even with the approval of the Guardians. As you will see by the recent advertisement for another nurse, no such duties are mentioned.

### A NEW OBJECTION TO STREET COLLECTIONS.

"A Correspondent" writes: I was pleased to see an article about collecting boxes in last week's Hospital, especially as my indignation had been much roused by the way in which money had been obtained for them in Kentish Town. I quite agree with you that it is a great mistake for children to go out with collecting boxes, but surely it is not so bad as for young women to stand about the streets stopping all the young men that pass them and offering to kiss them so many times in exchange for pennies. "Selling kisses" they called it, and the hospital boxes were used as an excuse for this conduct, which I consider disgusting, and I am sure a great many others will agree with me. I am aware of the fact that many of the women of to-day have forgotten that they are women, but I do think that "selling kisses" in the streets is going a little too far and ought to be put a stop to.

### PRIVATE NURSING.

"Nurse M. C. S. W." writes: I am a private nurse on my own account, and have not had a case for fourteen weeks, and the reason, in my opinion, is not far to seek. I read in an article in one of the London papers that there are five thousand ladies with independent incomes training for nurses in hospitals. If that is the case how are we, who have been nurses for the last sixteen or twenty years, to get our living? It is a shame that such a thing should be allowed in England. What are we to do if this still goes on? I know six deserving and good nurses who have to make way for ladies, who take the same fees we ought to have. If they were to nurse the poor there would be some humanity in it, but no, they have plenty of money in most cases, and want more. What are we to do? It seems to me there is nothing for us but the workhouse. Others can make a plea and get influential people to help them, but who helps us? Are there no benevolent people to help those who are so willing to help themselves? Could not some institution be opened for we older nurses who have much more experience than those younger ones, and who have already sacrificed ourselves many times for the suffering? When I say "older nurses" I mean women between 38 and 45, who are refused in institutions on account of age, I am sure there are many more in the same position as myself.

[No doubt our correspondent saw the statement referred to in print, but we think the writer of such a paragraph would find some difficulty in proving that anything approaching five thousand women of independent means are training with a view to superseding private nurses who work for their living.—Ed. *T. H.*]

### BED CAPES FOR MEN.

"A Superintendent of Nurses" writes from a parochial hospital: In answer to my letter a few weeks ago, asking about cloaks for male patients, you advised me to apply to the matron of the London Hospital for a pattern. I did so, and received a kind letter and a pattern cloak by return of post. As you take such a warm interest in parochial hospitals, you will be pleased to hear that the committee readily granted my request, and the men are wearing their red cloaks to-day for the first time. We are also having the beds in this hospital fitted up with spring mattresses, and shall do away with the old straw paliasses now in use. I thank you for the help you gave me in my difficulty.

[The writer's interest in the patients under her charge could not fail to secure the kindly co-operation of her committee. The example of this parochial hospital might well be emulated. We remember hearing of the offer of the gift of warm capes for use by the medical cases during examination in the O.P. department of a general hospital being refused because no one would be troubled to take charge of them.—Ed. *T. H.*]

### SPURIOUS CO-OPERATIONS AND HOMES.

"An Old Nurse" writes: I am amused by M. L. Canning's letter in last week's Hospital. The paragraph she takes to refer to her nurses' home (which I never heard of) I took to mean another co-operation, which it exactly describes, and from which nurses have often come to my sister's home, begging her, with tears of distress, to find them some work. So I suppose those who find anything in the paragraph that might apply will take it to themselves. I wonder nurses risk so much in these so-called co-operations without first ascertaining that they are really what they pretend to be.

## Figures and Tables

**Figure f1:**